# Evaluation of Enterprise Accounting Data Management Based on Maturity Model

**DOI:** 10.1155/2022/3891475

**Published:** 2022-09-05

**Authors:** Jun Xiang

**Affiliations:** Accounting Institute of Wuhan College, Wuhan 430212, China

## Abstract

The study of enterprise management driven by accounting data has not only great strategic significance but also great practical value and distinctive characteristics of The Times. Although the macro evaluation of DCMM is strong, its objective operability is slightly weak. Therefore, based on the maturity model, this article establishes a three-level accounting data management (AAM) capability evaluation index system, divides the maturity level and key process domains, and constructs an AAM capability evaluation model. Through the evaluation of the accounting data management ability of the case enterprise, the evaluation value of the AAM ability is calculated, and the measurement of the AAM ability is completed, which is helpful to improve the data management ability of the enterprise scientifically and efficiently.

## 1. Introduction

At present, China's economic development has entered a “new normal,” but economic development is closely related to the improvement of science and technology. With the nonstop improvement of science and innovation, information is a fundamental piece of it, which is rethinking the interaction and method of social administration and public key independent direction, an endeavor to board navigation, authoritative business cycle, and individual independent direction, and is becoming increasingly important for the development of modern enterprises and society [1]. Nowadays, accounting data is the top priority of data management and the huge commercial value, scientific research value, social management value, and the value of supporting scientific decision-making are being constantly recognized and developed. Scholars' research on AAM has gradually provided some improved overall ideas and strategies for optimizing AAM [2, 3]. However, at present, the domestic research is limited to a certain part of AAM or stays in the discussion of the relationship between the two, which cannot effectively combine the whole and part of AAM.

CMM (capability maturity model) refers to a staged description used by software development organizations to define, implement, measure, control, and improve their software processes [4], which is divided into five grades to evaluate software contracting ability to improve software quality. In recent years, scholars have introduced the maturity model into data management for research, thereby forming a data management CMM. Literature [5] put forward the maturity model of enterprise information portals and evaluates the maturity of the case enterprise information portal. Literature [6] applied the maturity model to the measurement of the integration of industrialization and industrialization, which enriches the theoretical methods of evaluating the integration of industrialization and industrialization. Literature [7] applied the maturity model to the evaluation of multiproject management level of the construction unit. Literature [8] applied the maturity model to divide enterprise informatization maturity into five stages: informatization preparation, information system introduction, integration and sharing, enterprise extension, and decision support.

Although CMM has certain advantages compared with other data management capability models, it can be better applied to organizations in China. However, according to the published documents at present, DCMM (data capability maturity model) evaluation is strong in macro but slightly weak in objective operability, and it does not classify the data. Moreover, there is no research on the combination of AAM and maturity in China at this stage. Therefore, aiming at the partial improvement of all aspects of AAM, this article establishes an evaluation model of AAM ability to promote the improvement of the overall ability, which has reference significance for the research of AAM among enterprises.

## 2. Theoretical Basis of Maturity Model

### 2.1. Overall Architecture

**Figure 1:**
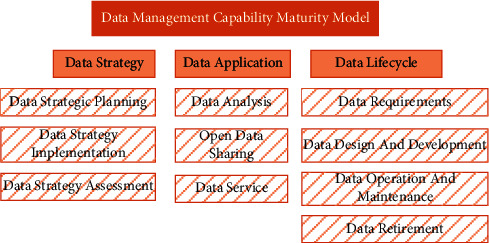
Architecture of DCMM.

As shown in Figure 1, three capability domains of data capability maturity evaluation are defined: data strategy, data application, and data life cycle. The biggest difference between DCMM and other data management capability maturity models is that DCMM increases the data application capability domain. At the same time, data strategy and data life cycle are two indicators that run through the application.

### 2.2. Grading

**Figure 2:**
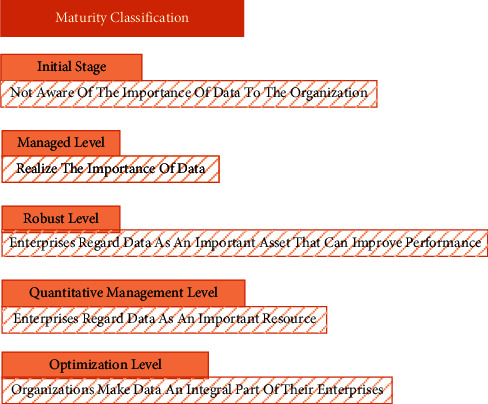
Grading of maturity.

Maturity is divided into five levels: initial level, managed level, steady level, quantitative management level, and optimization level [9, 10]. The positioning of each grade is shown in Figure 2:

 
*Grade 1*. Initial level: Organizations at this level are not aware of the importance of data to the organization, so they have not formed the consciousness of actively managing data. Data management within enterprises mainly runs through project management. No unified data management rules and procedures have been established within the enterprise, and the problems caused by the data lead to the low quality of customer service, which cost a lot of human resources to maintain the data.
 
*Grade 2*. Managed level: Organizations at this level realized the importance of data and began to manage data as an asset. Driven by the needs and strategies of the enterprise, the corresponding data management process was formulated, and the personnel within the organization were assigned to manage the data preliminarily, realizing and identifying the stakeholders of the data.
 
*Grade 3*. Robust level: Enterprises at this level regard data as an important asset that can improve performance. They have formulated organizational data management processes and policies to promote the standardization and standardization of data management. Data managers can manage across systems where the management of data can meet the requirements of the organization.
 
*Grade 4*. Quantitative management level: Enterprises at this level regard data as an important resource think that data management can enhance the competitiveness of enterprises and realize the promoting role of data in workflow and efficiency. All-round improvements have been made to the processes related to enterprise data management, performance indicators are set for relevant organizations, posts, personnel, etc., and they are regularly assessed so that the system and process can be optimized and improved according to the monitoring and analysis of data management, where data management is gradually becoming scientific and standardized.
 
*Grade 5.* Optimization grade: Organizations at this level regard data as an indispensable part of the enterprise. The policies and processes of AAM can be improved in real time according to the external environment and the current situation of the industry and are regarded as the benchmark of data management in the industry.

## 3. Enterprise AAM Evaluation Methods

### 3.1. Analytic Hierarchy Process

Analytic hierarchy process (AHP) is a multicriteria decision-making form combining quantitative and qualitative analysis. It is characterized in that based on analyzing the essence and internal relations of complex decision-making problems, a hierarchical structure model is constructed, and its process of complex problems is presented mathematically with less quantitative information. Through data to solve complex decision-making problems with multiobjectives, multicriteria, or no structural characteristics, the purpose of simplifying the decision-making scheme is achieved [11].

The general steps of AHP mainly include four steps. The first step is to build the hierarchical structure model; second, construct the judgment matrix; third, obtain hierarchical single sorting and its consistency check; and finally implement hierarchical general ranking and its consistency check.

#### 3.1.1. Establishment of a Hierarchical Structure Model

The decision to solve the problem is divided into three levels, namely, the target level, the decision criterion level, and the decision scheme level. In the application of the AHP, the problem to be solved is to calculate the relative weight of the bottom layer to the top layer, so as to sort the schemes and measures at the bottom layer and choose the best scheme [12, 13].

#### 3.1.2. Construction of a Comparative Judgment Matrix

The construction of judgment matrix is to determine the weight of each criterion layer to the target layer by comparing each element with each other pairwise. The comparative judgment matrix of *A* is as follows:

(1) $$\begin{array}{c} A={\left(a_{ij}\right)}_{m\times n}=\left(\begin{array}{cccc} a_{11} & a_{12} & \cdots & a_{1n}\\ a_{n1} & \cdots & \cdots & \cdots \end{array}\right)\text{,} \end{array}$$

where the elements in A should meet the following requirements: *a*_*ij*_ > 0; *a*_*ij*_=1/*a*_*ji*_; *a*_*ii*_=1.

#### 3.1.3. Hierarchical Single Sort

Hierarchical single sort refers to evaluating all elements in pairs for an element in the previous layer and arranging the important order. The concrete calculation can be carried out according to the judgment matrix A, and the calculation can ensure that it can meet the characteristic root and characteristic vector conditions of *AW* = *λ_ax_W*, where the largest feature root of *A* is *λ_ax_*, and the normalized feature vector corresponding to *λ_ax_* is *W*, and *W*_*i*_ is a component of *W*, which refers to the weight, and corresponds to the single ordering of its corresponding elements. Use the judgment matrix to calculate the weight of each factor *a*_*i*j_ to the target layer.

The calculation steps of the weight vector (*W*) and the maximum feature (*λ_ax_*) are as follows.

First of all, take the product of the row elements according to equation (2) and then raise it to the nth power:

(2) $$\begin{array}{c} {\overset{⟶}{W}}_{i}=\sqrt{\overset{n}{\underset{j=1}{\prod }}\;a_{ij}}\;i,j=1,2,\cdots ,n\text{.} \end{array}$$

Then, it is normalized into a ranking weight vector by formula (3), which is denoted as *W* (the element of *W* is the ranking weight of the relative importance of the factor in the same level to a factor of the previous level), then *W* = (W_1_, W_2_,…, W_n_)^T^ is the result of judging the hierarchical single ranking of the matrix.

(3) $$\begin{array}{c} W_{i}=\overset{n}{\underset{i=1}{\sum }}W_{i}\text{.} \end{array}$$

Finally, determine the maximum characteristic root of the matrix by the following formula:

(4) $$\begin{array}{c} \lambda_{\max}=\frac{1}{n}\overset{n}{\underset{i=1}{\sum }}\;AW_{i}\text{.} \end{array}$$

#### 3.1.4. Consistency Inspection and Hierarchical General Sorting

If the *n*-order judgment matrix is *B*, the maximum characteristic root λ_ax_ can be obtained by the following methods:

(5) $$\begin{array}{c} BW=\lambda W\text{.} \end{array}$$

The following consistency index CI is taken to test the consistency index of judgment:

(6) $$\begin{array}{c} CI=\frac{\lambda_{max}-n}{n-1}\text{.} \end{array}$$

CI = 0 means that the judgment matrix is completely consistent and the larger the CI, the more serious the inconsistency of the judgment matrix.

Assuming that *A* is the target layer, the weight coefficients of *m* total ranking of factor levels are as follows: *a*_1_, *a*_2_,…, *a*_*m*_; B is the middle layer, and the weight coefficients of *n* hierarchical single sort of factors are as follows: *b*_1_^1^ ⋯ *b*_*n*_^*m*^; therefore, the total ranking of layer B is calculated according to the following formula:

(7) $$\begin{array}{c} b_{i}=\overset{m}{\underset{j=1}{\sum }}\;a_{j}b_{ij}\text{.} \end{array}$$

Set the *B* layer *B*_1_, *B*_2_, ⋯, *B*_*n*_ On the upper layer (*A* Layer), the hierarchical ranking consistency index of factors *A*_*j*_(*j*=1,2, ⋯, *m*) is *CI*_*j*_, the random consistency index is *RI*_*j*_, and the consistency ratio of the hierarchical total sort is as follows:

(8) $$\begin{array}{c} CR=\frac{a_{1}CI_{1}+a_{2}CI_{2}+\cdots +a_{m}CI_{m}}{a_{1}RI_{1}+a_{2}RI_{2}+\cdots +a_{m}RI_{m}}=\frac{\sum a_{i}CI_{i}}{\sum \;a_{i}RI_{i}}=\frac{CI}{RI}\text{.} \end{array}$$

When <0.1, it is considered that the overall ranking of the hierarchy has passed the consistency test; otherwise, it is necessary to readjust the element values of the judgment matrix and make the final decision according to the overall ranking of the decision-making level.

### 3.2. Fuzzy Analytic Hierarchy Process

The fuzzy comprehensive evaluation method is an overall decision based on a single decision of all factors involved in things, which is a comprehensive evaluation affected by multiple factors. In contrast, the fuzzy analytic hierarchy process (FAHP) is an improved AHP, which combines the above AHP with the fuzzy comprehensive evaluation method. It is a comprehensive qualitative and quantitative evaluation model, which is suitable for dealing with uncertain problems. It is widely used in system evaluation, system optimization, efficiency evaluation, and so on.

Generally speaking, the first step is to use AHP to determine the weight of each index in the model index system, and the second step is to use the fuzzy comprehensive evaluation method to comprehensively evaluate the grade so as to effectively combine the two methods and obtain a more scientific evaluation result. The FAHP adopted in this paper is useful for studying the management ability of accounting data, which is applicable to determine the index weight, evaluate the maturity level, and establish the evaluation model of AAM ability.

## 4. Evaluation Model of Enterprise AAM Based on Maturity Model

### 4.1. Setting of Index System

**Figure 3:**
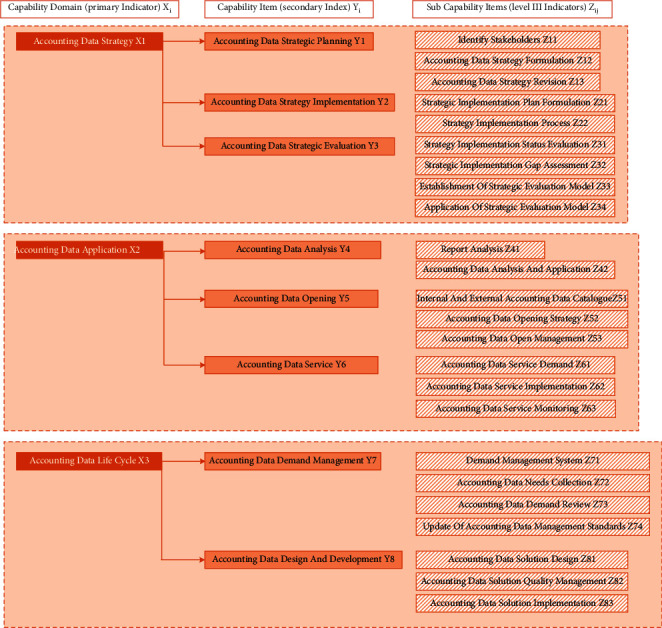
Distribution of index system.

The index system of the AAM capability evaluation model consists of three levels of indicators, including three competence domains (first-level indicators), eight competence items (second-level indicators), and 24 subcompetence items (third-level indicators). The weights of all levels of indicators in the AAM capability evaluation model are determined by AHP, and consistency tests are carried out in turn. The distribution of each indicator is shown in Figure 3.

#### 4.1.1. Accounting Data Strategy

Generally speaking, accounting strategy is to combine accounting data technology, concept, framework, and strategic management so as to build an accounting data analysis platform and enhance the overall core strength and environmental adaptability. There are three competency items under the accounting data strategy, which are accounting data strategy planning, accounting data strategy implementation, and accounting data strategy evaluation.

#### 4.1.2. Accounting Data Application

The application of accounting data is the process of mining effective information from accounting data using the method of accounting data analysis, providing users with auxiliary decisions, providing accounting data services, and maximizing the value of accounting data. There are three competency items in accounting data application: accounting data analysis, accounting data open sharing, and accounting data service.

#### 4.1.3. Accounting Data Life Cycle

The accounting data life cycle is the whole process of accounting data from design, development, creation, migration, application, archiving, recycling, reactivation, and withdrawal. There are four competency items in the accounting data life cycle: accounting data demand management, accounting data design and development, accounting data operation and maintenance, and accounting data retirement.

### 4.2. Maturity Level Setting

The maturity level of the AAM capability evaluation model is divided into five levels: initial level, repeatable level, defined level, managed level, and optimized level. Each maturity level has its own function. Except for the first stage, all the other stages are constructed according to the same internal structure. Different maturity levels reflect different levels of AAM capabilities.

#### 4.2.1. Initial Level

The initial level is characterized by passive management, and there is no initiative in AAM. The organization is not aware of the importance of AAM, and it is chaotic and disorderly as a whole, whose specific performance is that no standardized AAM policies, documents, plans, and processes have been formed. There is no formal AAM organization at the organizational level. There is no clear goal and practice. Moreover, there are no key process areas and key practices in this stage.

#### 4.2.2. Repeatable Level

**Figure 4:**
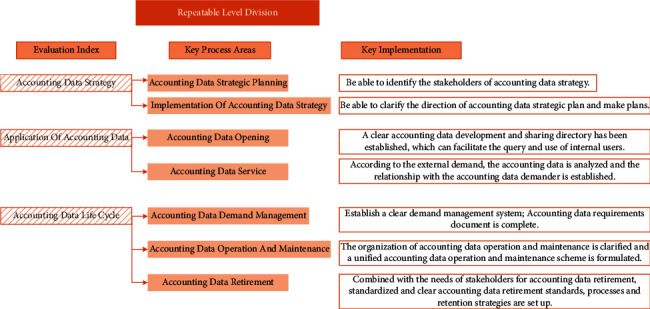
Division of repeatable level.

The repeatable level is the second stage of the accounting data management ability assessment model. Organizations in this stage initially realize the importance of accounting data management and begin to manage accounting data, but the scope of accounting data management is relatively limited. The definition of repeatable level is shown in Figure 4.

#### 4.2.3. Defined Levels

**Figure 5:**
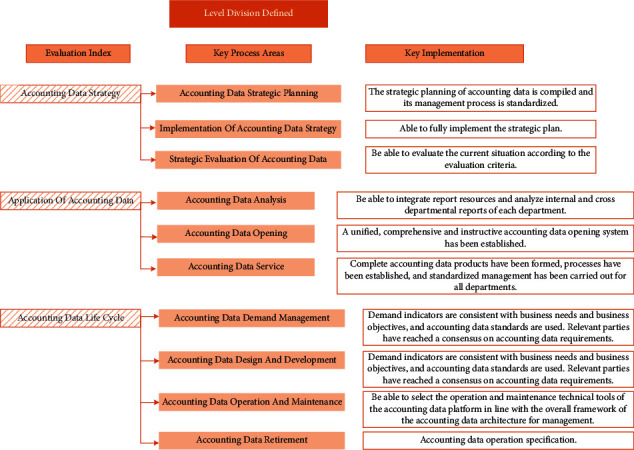
Division of defined level.

The third level of the AAM evaluation model is the defined level, which means that the key process areas of repeatable level have been realized, whose specific performance is as follows: the relevant system and process of AAM are more perfect and standardized; it is able to carry out comprehensive AAM within the organization; the role of AAM personnel has been enhanced. The specific distribution is shown in Figure 5.

#### 4.2.4. Managed Level

**Figure 6:**
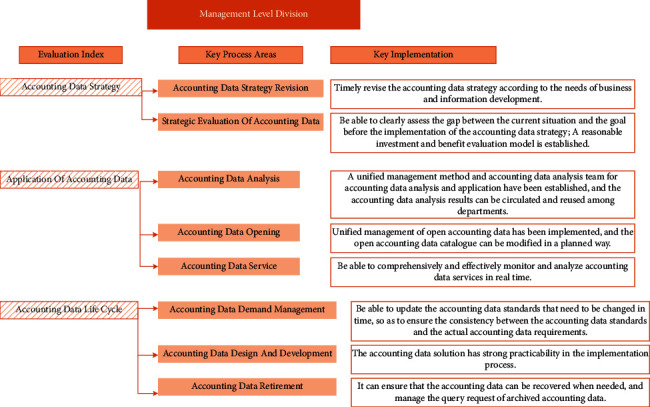
Division of managed level.

The fourth level of the AAM capability evaluation model is the managed level, which means that the key process areas of the defined level have all been realized. Specifically, it can optimize and update the systems and processes related to AAM; AAM is more systematic and professional, and its application in organizations is more standardized; AAM supports the decision-making of the organization and has made great progress. The specific distribution is shown in Figure 6:

#### 4.2.5. Optimized Level

**Figure 7:**
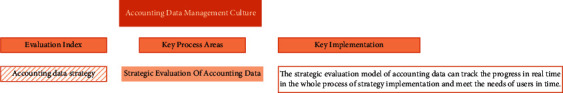
Division of optimized level.

The highest level of the accounting management capability evaluation model is the optimization level, which means that the key process areas of the managed level have all been realized. The concrete performance is as follows: all the basic construction, manpower reserve, material resources, and other aspects of support are ready; through the long-term practice of AAM, the organization has accumulated a lot of experience and can make timely adjustments to the changes of the internal and external environment; the AAM culture is formed. The specific distribution is shown in Figure 7:

### 4.3. Calculation of Maturity Level

#### 4.3.1. Fuzzy Evaluation Matrix

Before the fuzzy comprehensive evaluation of AAM ability, the evaluated fuzzy relationship should be expressed as a fuzzy evaluation matrix and then determine the evaluation set *A*={*A*_1_, *A*_2_, ⋯, *A*_*n*_}, in which *A*_*n*_ indicates the grade of the evaluated index. Because the maturity level is divided into five levels, in the evaluation set, *n*=5, the fuzzy evaluation matrix with 5 columns is set as follows

(9) $$\begin{array}{c} P=\left(\begin{array}{ccccc} p_{11} & p_{12} & p_{13} & p_{14} & p_{15}\\ p_{21} & p_{22} & p_{23} & p_{24} & p_{25}\\ \cdots & \cdots & \cdots & \cdots & \cdots \\ p_{j1} & p_{j2} & p_{j3} & p_{j4} & p_{j5} \end{array}\right)\text{,} \end{array}$$

where element *p*_*jn*_(*n*=1,2,3,4,5) refers to the probability that all the three-level evaluation indexes *Z*_*ij*_ included by a certain second-level index *Y*_*i*_ get n-level times, respectively, in the scoring, namely, the membership degree *p*_*jn*_ of the measured index, which is expressed by the following formula:

(10) $$\begin{array}{c} p_{jn}=\frac{The\;number\;of\;times\;the\;in\;dex\;Z_{ij}\;was\;graded\;n}{Total\;grading\;times}\text{.} \end{array}$$

Note: the secondary indicators are the same in the same matrix *Z*_*ij*_.

#### 4.3.2. Fuzzy Evaluation Matrix

The weight matrix of all the three-level evaluation indexes *Z*_*ij*_ contained in a certain two-level index *Y*_*i*_ is set as *Z*=(*z*_*i*1_, *z*_*i*2_, ⋯, *z*_*ij*_), where *Z*_*ij*_ represents the weight of the corresponding three-level index *Z*_*ij*_. Then, the fuzzy relationship evaluation matrix of indicators at all levels can be obtained by the calculation of the following formula:

(11) $$\begin{array}{c} M={\left(M_{ij}\right)}_{m\times n}=Z\times P\text{.} \end{array}$$

#### 4.3.3. Determine the Maturity Level

After the final fuzzy comprehensive evaluation result is obtained through the calculation in the previous section, the cascade of the enterprise's AAM ability can be calculated through the following formula:

(12) $$\begin{array}{c} L=M\times N\text{.} \end{array}$$

Among them, *N*={20,40,60,80,100},*M* = {Initial level, repeatable level, defined level, managed level, optimized level}.

According to the calculation results of *L*, the maturity level of AAM ability to which each first-level index and the final result belong can be determined. At the same time, in the process of grade judgment, the maturity grade of AAM ability can be judged according to the principle of maximum membership degree, and the judgment result can be further proved.

## 5. Case Analysis

Through the evaluation of the accounting data management ability of the case enterprise, the evaluation value of the accounting data management ability is calculated, and the measurement of the accounting data management ability is completed, which is helpful in improving the data management ability of the enterprise scientifically and efficiently.

### 5.1. Analysis of AAM

The evaluation of Company A was conducted by questionnaire. The respondents are mainly stakeholders of AAM in enterprise A, including department heads, managers, experts, and AAM professionals. According to the scores of the respondents on the related indicators of Company A, the basic information of each capability domain of Company A can be obtained, and the results are shown in Tables 1–3.

According to the calculation formula mentioned above, it can be concluded that the fuzzy relation evaluation vector of each first-level index is as follows:

(13) $$\begin{array}{c} \begin{array}{c} M_{x1}=\left(\begin{array}{ccccc} 0.0524 & 0.2467 & 0.3703 & 0.2972 & 0.0334 \end{array}\right),\\ M_{x2}=\left(\begin{array}{ccccc} 0.0619 & 0.1548 & 0.3763 & 0.2778 & 0.1292 \end{array}\right),\\ M_{x3}=\left(\begin{array}{ccccc} 0.0488 & 0.1331 & 0.4602 & 0.3108 & 0.0471 \end{array}\right). \end{array} \end{array}$$

By multiplying the weight vector composed of the weights of the first-level indexes with the above-mentioned first-level fuzzy relation evaluation matrix to obtain the final fuzzy relation evaluation vector, the following vector is obtained:

(14) $$\begin{array}{c} M_{x}=\left(\begin{array}{ccccc} 0.0503 & 0.1517 & 0.3807 & 0.3320 & 0.0853 \end{array}\right)\text{.} \end{array}$$

According to the principle of maximum membership degree, the maximum value in the vector is the third value, i.e., 0.3807, which can preliminarily determine that the AAM ability of Company A is defined.

### 5.2. Comprehensive Evaluation of AAM

**Figure 8:**
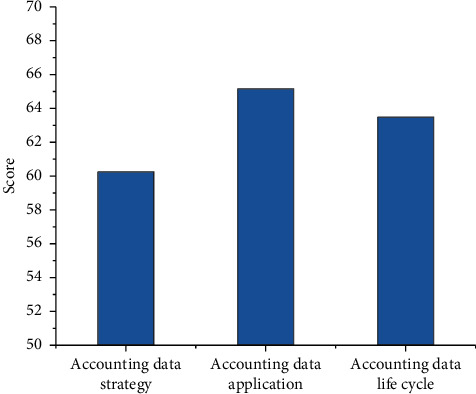
A Comprehensive evaluation of Company A's AAM ability.

Calculate the final evaluation score and grade of Company A's AAM ability, and the comprehensive evaluation results of enterprise AAM ability are shown in Figure 8:

The evaluation value of Company A's accounting data strategy is 60.25. Although the corresponding maturity level is the “defined level,” it is just entering the defined level, and there is still a big gap from the next level (managed level). Company A has not revised the accounting data strategy in time according to business and information development needs and cannot optimize the accounting data strategy in time. Before implementing the accounting data strategy, the gap between the present situation and the target cannot be clearly assessed. In addition, a reasonable investment and benefit evaluation model has not been established.

The evaluation value of Company A's accounting data application is 65.152. The corresponding maturity level is the “defined level,” and it is in the transitional stage between the defined level and managed level. It has not yet established a unified management method and accounting data analysis team for the application of accounting data analysis, and the results of accounting data analysis have not been circulated and reused among departments. In addition, we implement unified management of accounting data that has not yet been opened, which is failure to conduct comprehensive and effective monitoring and real-time analysis of accounting data services.

The evaluation value of the accounting data life cycle of Company A is 63.486. From the evaluation value, the corresponding maturity level is the “defined level,” and it is in the transitional stage between the defined and managed levels. Specifically, the demand indicators are consistent with the business needs and business objectives, accounting data standards are used, and relevant parties have reached a consensus on accounting data needs. Moreover, the solution meets the demand for accounting data, and the design content is complete; and the operation of accounting data retirement is standard.

## 6. Conclusion

Based on the maturity model, this article establishes a three-level evaluation index system of AAM ability, divides the maturity level and key process areas, and constructs the evaluation model of AAM. In addition, Company A, which has some experience in AAM, is selected to evaluate the effectiveness of the model. The evaluation values of accounting data strategy, accounting data application, and accounting data life cycle of Company A are 60.25, 65.152, and 63.486, respectively, and its AAM ability belongs to the “defined level.” The evaluation model of AAM ability established in this article can provide support for the research of AAM ability and provide guidance for the development of AAM ability evaluation.

## Figures and Tables

**Table 1:** Grading results of accounting data strategy.

First-level index	Second-level index	Third-level index	Grading				
			1	2	3	4	5
Accounting data strategy X1	Accounting data strategic plan Y1	Identify stakeholders Z11	1	5	11	13	0
		Strategy formulation Z12	0	3	12	14	1
		Revise strategy Z13	4	12	14	0	0
	Accounting data strategy implementation Y2	Implementation of strategic planning Z21	0	6	10	11	3
		Implementation process Z22	2	7	15	5	1
	Accounting strategy evaluation Y3	Evaluation of strategic implementation status Z31	2	8	12	8	0
		Implementation of strategic gap assessment Z32	0	7	9	12	2
		Establishment of strategic model Z33	6	15	7	2	0
		Evaluation model application Z34	5	16	7	2	0

**Table 2:** Grading results of accounting data application.

First-level index	Second-level index	Third-level index	Grading				
			1	2	3	4	1
Accounting data application X2	Accounting data analysis Y4	Analysis report Z41	0	0	10	15	5
		Analytical application Z42	0	1	9	11	9
	Accounting data opening Y5	Directory of Internal and External Accounting Data Z51	6	7	12	5	0
		Open strategy Z52	0	3	10	10	7
		Open management Z53	0	2	10	16	2
	Accounting data service Y6	Service Z61	4	11	14	0	1
		Implement service Z62	3	10	15	2	0
		Service Z63 monitoring	6	7	11	5	1

**Table 3:** Grading results of accounting data life cycle.

First-level index	Second-level index	Third-level index	Grading				
			1	2	3	4	1
Accounting data life cycle X3	Accounting data demand management Y7	Demand management system Z71	0	4	11	14	1
		Collect demand Z72	1	5	17	7	0
		Review requirements Z73	5	11	10	4	0
		Management standard update Z74	4	6	20	0	0
	Accounting data design and development Y8	Solution design Z81	0	5	21	4	0
		Solution Quality Management Z82	1	2	18	8	1
		Solution implementation Z83	0	1	7	21	1

## Data Availability

The dataset can be accessed upon request.
